# Human-Robot Interaction With Robust Prediction of Movement Intention Surpasses Manual Control

**DOI:** 10.3389/fnbot.2021.695022

**Published:** 2021-09-30

**Authors:** Sebastijan Veselic, Claudio Zito, Dario Farina

**Affiliations:** ^1^Department of Clinical and Movement Neurosciences, University College London, London, United Kingdom; ^2^Wellcome Centre for Human Neuroimaging, University College London, London, United Kingdom; ^3^School of Computer Science, University of Birmingham, Birmingham, United Kingdom; ^4^Autonomous Robotics Research Centre, Technology Innovation Institute, Abu Dhabi, United Arab Emirates; ^5^Department of Bioengineering, Imperial College London, London, United Kingdom

**Keywords:** physical human robot interaction, motion intention estimation, motion prediction, AI assistance, reach and grasp

## Abstract

Physical human-robot interaction (pHRI) enables a user to interact with a physical robotic device to advance beyond the current capabilities of high-payload and high-precision industrial robots. This paradigm opens up novel applications where a the cognitive capability of a user is combined with the precision and strength of robots. Yet, current pHRI interfaces suffer from low take-up and a high cognitive burden for the user. We propose a novel framework that robustly and efficiently assists users by reacting proactively to their commands. The key insight is to include context- and user-awareness in the controller, improving decision-making on how to assist the user. Context-awareness is achieved by inferring the candidate objects to be grasped in a task or scene and automatically computing plans for reaching them. User-awareness is implemented by facilitating the motion toward the most likely object that the user wants to grasp, as well as dynamically recovering from incorrect predictions. Experimental results in a virtual environment of two degrees of freedom control show the capability of this approach to outperform manual control. By robustly predicting user intention, the proposed controller allows subjects to achieve superhuman performance in terms of accuracy and, thereby, usability.

## 1. Introduction

Automation is leading to major societal changes, with an estimated 50% of current jobs being subjected to automation (Benedikt Frey and Osborne, 2017). However, automation often requires intelligent semi-autonomous robots operated by human users with physical human-robot interaction (pHRI) (De Santis et al., 2008). For example, there is a growing need for efficient controllers for robot manipulators involved in operations such as nuclear waste disposal or manufacturing processes (Marturi et al., 2016). For these devices, algorithms to predict user intention are a crucial component of the control system, as the purpose of pHRI is to achieve the planned goals of the user in a given domain (Losey et al., 2018).

**Figure 1:**
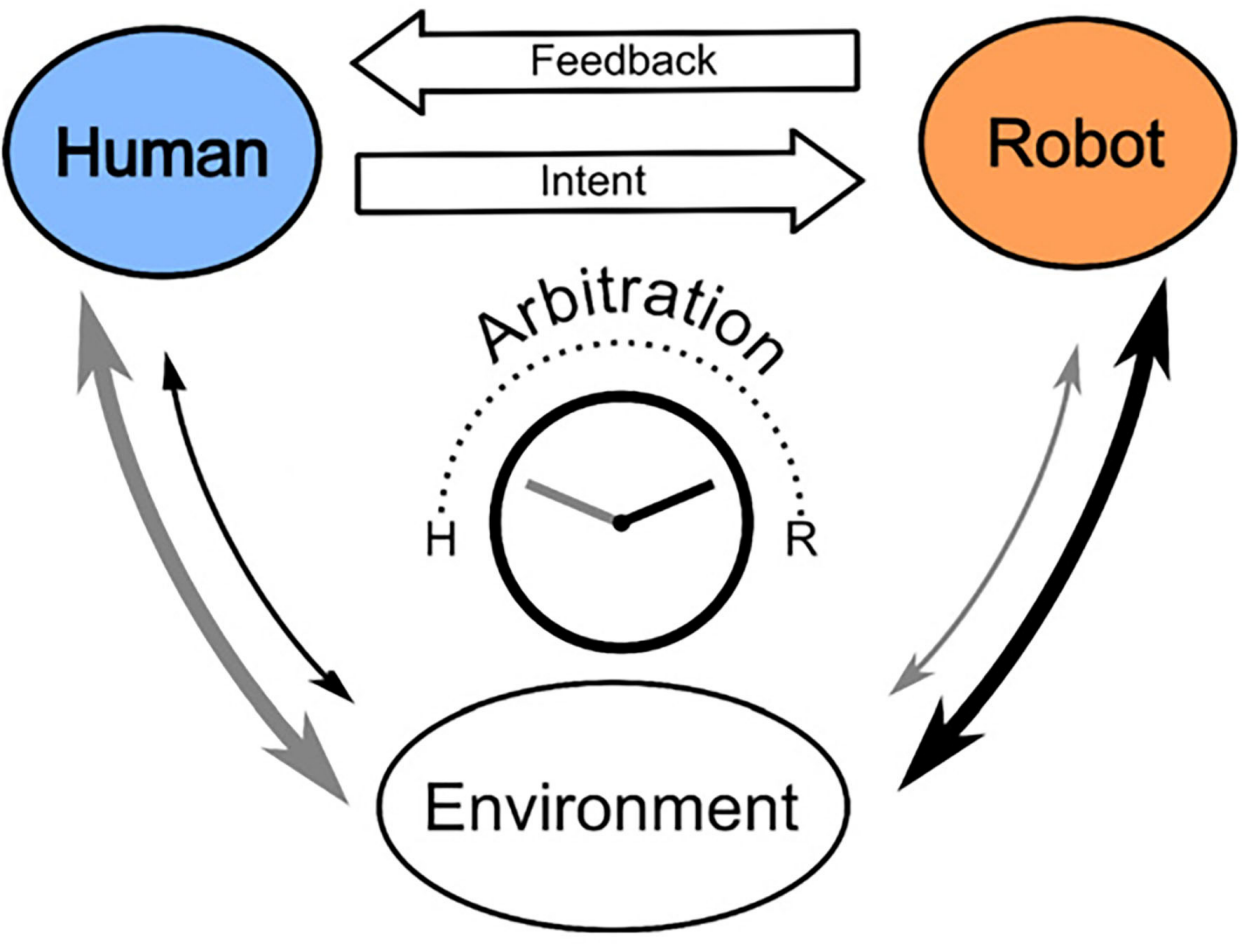
Adapted from Losey et al. (2018). A scheme of the interplay between robot feedback and human intention in physical human-robot interaction (pHRI). Humans receive feedback from the robot (controller) system once it decodes human task-specific intention. The robot and the human jointly exert control on the environment where the degree of control from either is determined through arbitration for a given domain.

If controller systems employed in pHRI settings could infer movement intention, this would better support the achievement of planned goals (Figure 1). In the scheme of Figure 1, the user sends an intention that is intercepted by a controller. The controller decodes that intention in a given task and sends (corrective) feedback to the user. In this study, the intention may be decoded from myoelectric signals generated by the muscles of the user, or from the movement kinematics of the user which encodes their intention of moving to a particular location. To complete the loop, the controller and the user jointly influence the interaction with the environment when their actions are modulated according to an arbitration process (Losey et al., 2018).

In this study, a novel way of achieving this pHRI coupling was developed by enhancing the robot component with context- and user-awareness. Context-awareness enables the system to perceive the environment and to identify potential actions for the user. User-awareness enables the system to recognize the intention of a user and assist them in their planned movement. The central contribution of this study is achieving high accuracy in reaching tasks by including user- and context-awareness in a pHRI system.

Context-awareness was enabled by a pre-defined set of candidate grasp targets and their reach-to-grasp trajectories for the testing scenario representing a scene with multiple graspable objects. This set of candidate reach-to-grasp trajectories represents the optimal trajectories to reach these target states (i.e., graspable objects) from a starting state, and they represent the input to the controller.

User-awareness was implemented through a time-variant Linear-Quadratic Regulator (LQR) controller (Kwakernaak and Sivan, 1972; Li and Todorov, 2004) (TV-LQR) that filters the motion commands of a user at each time step and assists the user along the trajectories of the candidate grasps, thereby inferring movement intention.

The controller proposed in this study extends the TV-LQR to deal with several trajectories at the same time. For each waypoint belonging to any of the target trajectories, the feedback controller proposes to either follow the current trajectory or move toward a neighboring one. The proper feedback control law is selected online by filtering the motion input of the user. If the user recognizes that the system is moving toward an incorrect target, the user is expected to apply a corrective motion that is used to compute the feedback control to guide the system toward a new best candidate. The TV-LQR is ideally suited for such computations as it can deal with potential changes in system dynamics and user input by computing optimal corrective feedback for all points on a given trajectory (Li and Todorov, 2004), without the need for predefined thresholds for the switching conditions.

Our proposed method can be distinguished from previous study in several ways. First, contrarily to existing methods for tele operation that mostly rely on crude force feedback *via* a haptic device, (e.g., Boessenkool et al., 2013; Narayanan et al., 2016), we proposed a predict-then-blend approach, in which the most likely intention of the user is estimated first, followed by assistance being provided in the tested task. The previous study related to intention detection and user assistance typically relies on myoelectric or EEG recordings which have limitations associated with a low signal-to-noise ratio (reviewed in Lobo-Prat et al., 2014; Losey et al., 2018). Conversely, we estimate the motor intention directly from the user kinematics, which has been shown to be effective in our previous study (Heiwolt et al., 2019). Furthermore, LQR controllers have previously been employed for solving the problem of user assistance (Borner et al., 2015) and correcting user-given input (Medina et al., 2012; Moualeu and Ueda, 2014). Existing approaches aiming to predict user intention are based on computing the distance between the current configuration of the user and the desired one (Narayanan et al., 2016). However, such approaches cannot scale to complex and dynamic environments or tasks because they ignore the history information and kinematic limitations of the tele operated robot.

In addition to predicting user intention, shared control involves using the predictions to support the user in achieving the expected goal. In this study, we proposed an approach that enables the soft integration of input commands and predictions. The user constantly provides inputs to the system *via* semi-autonomous control and the TV-LQR estimates the most likely input at each time step by simply filtering the commands of the user. If the user is following along the planned trajectory, the commands are filtered to follow it. Otherwise, the feedback controller dilutes its adjustment and enables the user to smoothly transition to the closest planned trajectory.

In summary, a generic formulation for detecting the motion intention of the user based on the TV-LQR to filter and optimize human motor control in a grasping scenario is proposed. Moreover, using a predictive extension of the TV-LQR, the desired goal of the user is predicted and inferred in an unsupervised manner. Finally, the controller is tested and compared to manual control, as an important aspect of pHRI will be superhuman performance according to at least one objective function (e.g., accuracy, computational cost, or speed) (Van Den Berg et al., 2010), which will facilitate the adoption of these approaches in practice.

## 2. Methods

The proposed TV-LQR implementation is part of a robot grasping framework previously described in Kopicki et al. (2014) and Zito et al. (2019). In this study, we present an important innovation of this framework that enables a human user to control the position of the robot arm using a control input device (e.g., keyboard, joystick, or haptic device) which is filtered in such a way as to always keep the state of the robot arm along the trajectory of the user target goal. In this way, we translate the TV-LQR implementation to a pHRI scenario. When presented with a scene (e.g., a table with objects available for grasping such as a kettle or a bottle), the grasping framework can create optimal trajectories for potentially feasible grasps for the mentioned objects from a given starting pose (i.e., the initial position of the robot arm). It is then able to execute a given trajectory and grasp a specific object. In the current extension, if the scene has several graspable objects, the controller would support the user to follow one optimal trajectory based on the prediction of the intention of the user. Importantly, this extension allows the user to switch the target goal with the controller accordingly exerting less corrective force (Supplementary Figures 1, 2). The continuous corrective feedback from the controller is received until the pose of the system reaches the target goal of the trajectory. At this position, the grasping mechanism implemented within the grasping framework would become active and grasp the target object. The experimental setup aims to demonstrate that our proposed framework can be used to improve reach-to-grasp performance in tele operated systems by robustly predicting movement intention.

### 2.1. Formal Characterization of the State-Space Model and TV-LQR

The implementation of the TV-LQR is described in terms of a discrete, state-space model:


(1) $$\begin{array}{r} {\text{x}}_{\left(t+1\right)}=A_{\left(t\right)}{\text{x}}_{\left(t\right)}+B_{\left(t\right)}{\text{u}}_{\left(t\right)} \end{array}$$

Where **x** = [*x, y, z*, ϕ, ψ, θ]^⊤^∈ℝ^6^ represents the system state and $\text{u}={\left[ẋ,ẏ,ż,\overset{∙}{\phi },\overset{∙}{\psi },\overset{∙}{\theta }\right]}^{⊤}\in {\mathbb{R}}^{6}$ represents the control input as state and angle velocities. All subscripts (i.e., (*t*) and (*t*+1)) represent time notation represented as discrete time steps. The passive transition dynamics are given by $A_{\left(t\right)}\in {\mathbb{R}}^{6\times 6}$, which in our chosen case scenario is kept constant over time with the following structure:


$$A=\left[\begin{array}{cccccc} 1 & -0.01 & 0 & 0 & 0 & 0\\ -0.01 & 1 & 0 & 0 & 0 & 0\\ 0 & 0 & 1 & 0 & 0 & 0\\ 0 & 0 & 0 & 1 & -0.01 & 0\\ 0 & 0 & 0 & -0.01 & 1 & -0.01\\ 0 & 0 & 0 & 0 & -0.01 & 1 \end{array}\right]$$

Such a parameterization leads to a small amount of damping in both the *x* and *y* direction and the ψ and θ angles. A simplifying assumption that was made was linear dynamics for the *z*-direction to make the system continuously move along one of the axes to simulate movement toward the target goals. Finally, $B_{\left(t\right)}\in {\mathbb{R}}^{6\times 6}$ represents the control matrix and filters how strongly the input of the user affects the updating of the overall system. The *B*_(*t*)_ is structured as follows:


$$B=\left[\begin{array}{cc} B_{11} & B_{12}\\ B_{21} & B_{22} \end{array}\right]$$

where $B_{11}=I\in {\mathbb{R}}^{3\times 3}$ and the rest of the blocks are zero 3 ×3 matrices, reducing the input of the user command to only control translations. In the testing scenario, for each target object *j*, a trajectory $\left[{\text{x}}_{0}^{j},{\text{u}}_{0}^{j},{\text{x}}_{1}^{j},{\text{u}}_{1}^{j},\ldots ,{\text{x}}_{j_{n}}^{j}\right]$ was generated as a set of waypoints and optimal control inputs, such that each **x**^*j*^, **u**^*j*^ are subject to the system dynamics defined in Equation (1).

Using the state space formulation above, at each time step the current position of the system **x**_(*t*)_ and input **u**_(*t*)_ are recomputed w.r.t. the reference frame of the best matching waypoint for each trajectory, such that


(2) $$\begin{array}{r} {\;}^{j}{\hat{\text{x}}}_{\left(t\right)}={\text{x}}_{\left(t\right)}-{\text{x}}_{\left(t\right)}^{j}\\ {\;}^{j}{\hat{\text{u}}}_{\left(t\right)}={\text{u}}_{\left(t\right)}-{\text{u}}_{\left(t\right)}^{j} \end{array}$$

where the notation ${\;}^{j}{\hat{\text{v}}}_{\left(t\right)}$ represents a vector **v**_(*t*)_ at time *t* seen from the *j* trajectory. The best matching waypoint for any trajectory *j* at time step *t*, ${\text{x}}_{\left(t\right)}^{j}$, is computed as the closest waypoint ${\text{x}}_{l}^{j}$ to **x**_(*t*)_. Note that the subscription (*t*) identifies the time step for the TV-LQR system, while *l* identifies the waypoint along the trajectory.

The obtained estimates from Equation (2) are used at each waypoint to compute the quadratic cost of the current system state and input:


$$J_{\left(t\right)}^{j}{=}^{j}{\hat{x}}_{\left(t\right)}^{⊤}Q_{\left(t\right)}{\;}^{j}{\hat{x}}_{\left(t\right)}{+}^{j}{\hat{u}}^{⊤}R_{\left(t\right)}{\;}^{j}{\hat{u}}_{\left(t\right)}$$

The *Q* and *R* cost matrices are defined as:


$$\begin{array}{lll} Q & = & \left[\begin{array}{cccccc} e^{\frac{S}{1+t\tau }} & 0 & 0 & 0 & 0 & 0\\ 0 & e^{\frac{S}{1+t\tau }} & 0 & 0 & 0 & 0\\ 0 & 0 & e^{\frac{S}{1+t\tau }} & 0 & 0 & 0\\ 0 & 0 & 0 & e^{\frac{S}{1+t\tau }} & 0 & 0\\ 0 & 0 & 0 & 0 & e^{\frac{S}{1+t\tau }} & 0\\ 0 & 0 & 0 & 0 & 0 & 1 \end{array}\right]\\ R & = & \left[\begin{array}{cccccc} e^{\frac{S}{1+t\tau }} & 0 & 0 & 0 & 0 & 0\\ 0 & e^{\frac{S}{1+t\tau }} & 0 & 0 & 0 & 0\\ 0 & 0 & e^{\frac{S}{1+t\tau }} & 0 & 0 & 0\\ 0 & 0 & 0 & e^{\frac{S}{1+t\tau }} & 0 & 0\\ 0 & 0 & 0 & 0 & e^{\frac{S}{1+t\tau }} & 0\\ 0 & 0 & 0 & 0 & 0 & e^{\frac{S}{1+t\tau }} \end{array}\right] \end{array}$$

Where the *Q* matrix represents penalization for being away from the optimal trajectory while the *R* matrix represents how strongly a divergence of the user input against the optimal input is penalized. It is worth emphasizing is that the diagonal terms of both matrices are parameterized over time with a hyperbolic discounting function (Supplementary Figure 1):


(4) $$\begin{array}{r} f\left(S,t\right)=e^{\frac{S}{1+t\tau }} \end{array}$$

where the parameter τ is a constant reflecting discounting strength of the scaling matrix. The values for *S* and τ are empirically estimated in section 3 *via* our characterization run experiments.

By minimizing the cost function in Equation (3), the expected trajectory that the user wants to follow is found as the closest to the current system state. We denote the selected waypoint of the chosen trajectory as ${\text{x}}_{\left(t\right)}^{j*}$ and ${\text{u}}_{\left(t\right)}^{j*}$ and we denote the state and input of the system w.r.t. the trajectory


$$\begin{array}{c} {\hat{\text{x}}}_{\left(t\right)}={\text{x}}_{\left(t\right)}-{\text{x}}_{\left(t\right)}^{j*}\\ {\hat{\text{u}}}_{\left(t\right)}={\text{u}}_{\left(t\right)}-{\text{u}}_{\left(t\right)}^{j*} \end{array}$$

as already defined in Equation (2) but for simplicity, we drop the reference to the selected trajectory j^*^.

We can now predict where the system state will be at the next time step given the current selected trajectory and the input of the user (${\hat{\bar{\text{x}}}}_{\left(t+1\right)}$):


(5) $$\begin{array}{r} {\hat{\bar{\text{x}}}}_{\left(t+1\right)}=A_{\left(t\right)}\hat{{\text{x}}_{\left(t\right)}}+B_{\left(t\right)}{\hat{\text{u}}}_{\left(t\right)} \end{array}$$

In addition, a conservative estimate heuristic of where the user wants to move next together with a two-waypoint trajectory buffer was added to the formulation. For example, if the user was at waypoint *t* = 15 and the position of the trajectory waypoint with the lowest cost was determined to be ${\;}^{k}{\text{x}}_{\left(16\right)}$ (i.e., trajectory *k* at waypoint *t* = 16), but the last two trajectory waypoints were on trajectory *h*, the latter was then picked. However, the trajectory with the lowest cost (*k*) was put in a temporary buffer. Once it happened that in two consecutive observations, the lowest cost trajectory was the same and not the one for which the user would later receive feedback, that trajectory was picked (Supplementary Figure 3). The utilized heuristic was more important for initial waypoints with a small distance between neighboring trajectories. Over time it became less important as no crossing appeared in the scenarios presented in this study. However, to decrease feedback strength when the user wants to switch trajectories, the scheme presented in Supplementary Figure 2 was employed.

Once the output of the Equation (5) is obtained, the optimal input ${\hat{\text{u}}}_{\left(t\right)}^{*}$ (Equation 6) can be computed where the term is still formulated w.r.t. to the corresponding ${\text{u}}_{\left(t\right)}^{j}$.


(6) $$\begin{array}{r} {\hat{\text{u}}}_{\left(t\right)}^{*}=\alpha {\hat{\text{u}}}_{\left(t\right)}-\left(1-\alpha \right)K_{\left(t\right)}{\hat{\bar{\text{x}}}}_{\left(t+1\right)} \end{array}$$

**Figure 2:**
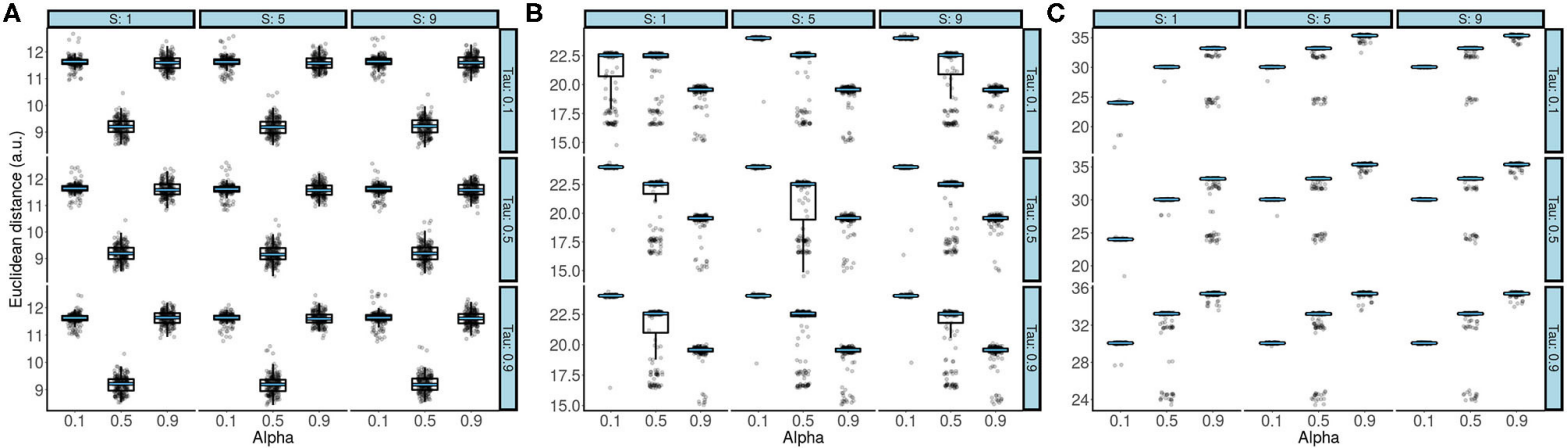
Boxplots overlaid with raw data showing the effect of (τ, α, *S*) on the Euclidean distance after passing 50 trajectory waypoints **(A)**, 100 trajectory waypoints **(B)**, and 200 trajectory waypoints **(C)**. The black line of the box plot denotes the median value. The upper and lower bound of the boxplot denotes the 1st and 3rd quartile of the dataset. The boxplot whiskers denote the 1.5* Inter quartile range (IQR). The superimposed blue line on the boxplots denotes the median and has been added for clarity purposes. In all three panels, the α parameter had the strongest effect on Euclidean distance.

Equation (6) is critical to the updating process and, therefore, to the overall system behavior (i.e., following the nominal trajectory or moving toward a more promising one). It introduces a constant parameter α, providing similar functionality to the Kalman gain (Kalman, 1960). This parameter arbitrates between weighting the pure user input (${\hat{\text{u}}}_{\left(t\right)}$) and the state prediction (${\hat{\bar{\text{x}}}}_{\left(t+1\right)}$) filtered by the feedback matrix (*K*_(*t*)_) (as shown in Figure 2). This means that high α values would lead to strong discounting of the feedback matrix filtering, thereby making the optimal input more dependent on user input. In contrast, low values would lead to strong discounting of the user input and would, therefore, favor the filtered state information for the optimal input. To compute *K*, the finite-horizon, discrete-case of LQR was used at each waypoint of each trajectory; corresponding to each change of the scaling of the *Q* and *R* matrix


(7) $$\begin{array}{r} K_{\left(t\right)}={\left(R+B^{⊤}P_{\left(t+1\right)}B\right)}^{-1}\left(B^{⊤}P_{\left(t+1\right)}A+N^{⊤}\right) \end{array}$$

Importantly in the equation above, by computing *P*_(*t*)_ which is obtained by solving the finite-horizon, discrete-case Algebraic Riccati equation (Equation 8)


(8) $$\begin{array}{r} P_{\left(t-1\right)}=A^{⊤}P_{\left(t\right)}A-\left(A^{⊤}P_{\left(t\right)}B+N\right){\left(R+B^{⊤}P_{\left(t\right)}B\right)}^{-1}\\ \;\left(B^{⊤}P_{\left(t\right)}A+N^{⊤}\right)+Q \end{array}$$

we minimize the cost function:


(9) $$\begin{array}{r} J={\text{x}}_{\left(N\right)}^{⊤}Q_{\left(N\right)}{\text{x}}_{\left(N\right)}+\overset{N-1}{\underset{t=0}{\sum }}\left({\text{x}}_{\left(t\right)}^{⊤}Q_{\left(t\right)}{\text{x}}_{\left(t\right)}+{\text{u}}_{\left(t\right)}^{⊤}R_{\left(t\right)}{\text{u}}_{\left(t\right)}\right) \end{array}$$

With ${\text{x}}_{N}^{⊤}$ being the final goal state for the j^*^ trajectory. Finally, the obtained optimal control estimate is dereferenced to obtain the optimal input (${\text{u}}_{\left(t\right)}^{*}$) that is then continuously supplied to the system as the control input.


(10) $$\begin{array}{r} {\text{u}}_{\left(t\right)}^{*}={\hat{\text{u}}}_{\left(t\right)}^{*}+{\text{u}}_{\left(t\right)}^{j} \end{array}$$

After the optimal input was computed, the prediction estimate of the system pose was obtained using the model from Equation (1) with a substitution in the input term (Equation 11) and the corresponding local optimal trajectory state estimate being used in the state term:


(11) $$\begin{array}{r} {\hat{x}}_{\left(t+1\right)}=A_{\left(t\right)}{\hat{\text{x}}}_{\left(t\right)}+B_{\left(t\right)}{\text{u}}_{\left(t\right)}^{*} \end{array}$$

In the last step, the state vector was dereferenced w.r.t. to the local optimal trajectory and put back into the world frame:


(12) $$\begin{array}{r} {\text{x}}_{\left(t+1\right)}={\hat{\text{x}}}_{\left(t+1\right)}+{\text{x}}_{\left(t\right)}^{j} \end{array}$$

To this state prediction, mean-centered, Gaussian noise with σ = 1 was added to both the *x* and *y* coordinate to approximate noisy updates due to faulty odometry or sensor readings:


(13) $$\begin{array}{r} {\text{x}}_{\left(t+1\right)}={\text{x}}_{\left(t+1\right)}+N\left(0,\sigma \right) \end{array}$$

### 2.2. Experimental Tests

#### 2.2.1. Scenario

The presented system was tested in a scenario where multiple trajectories were generated from an arbitrary starting point representing the starting system pose *x*_0_. The generated trajectories represented potential grasp targets (Supplementary Figure 4), and were described to the subjects as optimal trajectories from their starting system pose to the potential grasp targets, as outlined in the Procedure. On each trial, each subject was first tasked with providing their desired target grasp (i.e., the name of its corresponding trajectory). This was followed by them continuously providing input to the system by means of a wired computer mouse, with input sampled at 10 Hz. As they provided input, the pose of the system was updated.

#### 2.2.2. Procedure

The implementation was tested in two parts. The first part is referred to as characterization runs throughout this manuscript. The second part is referred to as controller tests. In both cases, control was supplied using a wired computer mouse and calibrated on a computer screen using a 1,920 ×1,080 resolution, where the coordinate (20, 1,020) would represent *u* = [0, 0, 1, 0, 0, 0]^⊤^. By moving the mouse along the *x* and *y* axis in physical space, one could increase or decrease the velocity of the respective dimension, while the *z*-axis would slowly be increased to mimic moving from a starting system pose toward a target grasp pose in one dimension. The goal of the task was to go from the starting system pose toward a target grasp pose. In the first part, 200 trials were used for each parameter combination to measure their effect on the Euclidean distance between the final system pose and target grasp pose (i.e., accuracy). The model space for the employed was: τ (0.1, 0.5, 0.9), *S* (1, 5, 9), and α (0.1, 0.5, 0.9). Additionally, trajectory lengths were varied as well with 50, 100, and 200 waypoints through the characterization runs. A given parameter or employed trajectory length was varied individually while the rest of the test characteristics were kept constant, for each possible parameter combination this was repeated 200 times. Furthermore, Gaussian noise (σ) was set to a constant value (σ = 1). In general, in characterization runs, constant input was provided by keeping the mouse position (i.e., the velocity) constant beginning with a starting pose until the vicinity of the target grasp pose was reached. At that stage, the Euclidean distance between the final system pose the and closest target grasp pose was computed. In this section, the constant user input was simulated as it would have been unfeasible for human users to perform trials with all possible parameter combinations.

While the characterization results showed that α = 0.1 was the optimal parameter for a trajectory length of 200 waypoints, α = 0.5 was used to enable equal arbitration between feedback provided by the TV-LQR and user-provided control in the TV-LQR condition (Equation 7).

In the second part, the described setup was tested on six subjects aged between 24 and 27 years with no history of movement disorders and complete upper-limb motor mobility to avoid unwarranted issues in operating the system. Before starting, subjects were told they would need to provide input to guide a controller in a simulated 3D scene from a starting system pose to a final system pose that would be as close as possible to one of the target grasp poses. Furthermore, they were told there were several possible target grasps in the simulated scene, representing possible grasp candidates. Finally, they were told there was an optimal trajectory from a given starting pose to a target grasp pose which the TV-LQR would try to keep them as close as possible to in one of the conditions they were about to be tested on.

To enable navigation through this, simulated 3D scene subjects were presented with information of their current position in terms of *x*, *y*, and *z* coordinates, their velocity, the coordinates of several final target grasp poses, the waypoints of the optimal trajectories closest to their current position, and the coordinates of the trajectory they were closest to, in case it was not one of the target ones.

In contrast to characterization runs, the goal of each subject in the second part was to pick one of three target grasp poses that were arbitrarily defined at the start of each trial and provide input to move the controller such that they would achieve a final system pose which would be as close as possible to their target grasp pose (i.e., the final pose of a specific trajectory). In controller tests, they were required to switch their targeted grasp half way through the execution (switch condition) as opposed to the other half, where this was not necessary (non-switch). In both the switch and non-switch conditions subjects were required to focus on, arbitrarily chosen, target grasp poses from three trajectories (F, I, J).

Therefore, an optimally accurate system would always lead to either of these three trajectories and none of the remaining trajectories (A–E, G, and H). This factor was orthogonal to the assisted and manual condition. That is, to the condition where they were assisted by the TV-LQR when providing input (assisted) and the one where their final system pose dependent entirely on their performance (manual). All subjects were tested on a trajectory length of 200 waypoints and using 100 trials per each of the described conditions, amounting to 200 trials per subject using the optimal parameter estimates from the characterization runs. For each subject, this experiment took between 60 and 90 min.

#### 2.2.3. Analysis

The analysis comprised two parts. In the first part, individual conditions of interest from the characterization tests of the system were compared either using Wilcoxon rank sum tests or 2-sided, independent-samples Welch *t*-tests to establish optimal parameter settings. Only accuracy was tested, as the speed of execution was not stored.

In the second part, further Welch *t*-tests and Wilcoxon rank sum tests were performed to compare the TV-LQR assisted and manual condition as a whole and segregated in the non-switch and switch condition to assess whether any changes were observed. This provided a direct test for the hypothesis of the TV-LQR assisted condition leading to higher accuracy. As a more stringent criterion, linear mixed models (LMM) were employed to factor in the repeated-measured aspect of the design. In addition, for each subject, a linear regression on the complete dataset collapsed across the switch and non-switch condition was performed to assess whether the slope (β) coefficient from the assisted to the manual condition would be positive. Namely, this would indicate that for all subjects, the same results hold, and our results were not driven by outliers (e.g., one subject in the dataset). Finally, the results were inspected to assess whether in the TV-LQR condition, subjects were led to their desired trajectories and how this compared to manual control where no assistance was provided. In this study, the final goal states closest to the system pose were plotted as histograms of visitation frequency and checked to assess whether there was a saturation of visitations for the trajectories F, I, and J and whether the pattern of visitations differed between the conditions.

## 3. Results

The controller was first tested in simulation through characterization runs to determine the optimal parameter set (τ, α, *S*) for the testing domain and to investigate their impact on accuracy (as shown in section 4). In this study, accuracy was defined as the Euclidean distance between the final system pose and the grasp target pose for a specific target trajectory after 50, 100, and 200 trajectory waypoints under constant input. The final pose was always an arbitrarily picked grasp target pose of a specific trajectory that remained the same throughout an execution run. By keeping the input and final pose constant, the contribution of individual parameters (τ, α, *S*) on accuracy was determined. Crucially, despite providing constant input and a constant final pose, non-additive unit noise (Equation 14) was added at each waypoint for each execution run, and thus, all reported results include noise because the aim was to design a robust control system that would generalize to real-world domains.

The optimal parameter set was then used on human subjects where the controller was directly compared to manual control with a within-subjects design. Due to a within-subjects design, the sample size was (*N* = 6) subjects and is comparable to previous related study (Shamaei et al., 2015; Tao et al., 2017; Sierra et al., 2019).

### 3.1. Characterization Runs on Trajectories With 50 Waypoints

The characterization run results with 50 waypoints are reported first (Figure 2A). Two independent-sample Welch *t*-tests showed that the parameter setting α = 0.5 (*M* = 9.20±0.33) yielded best accuracy compared to both α = 0.1 [*M* = 11.62±0.23, *t*_(3427.5)_ = 244.45, 95% CI [2.40, 2.44], *p* = 2.2*e*-16] and α = 0.9 [*M* = 11.59±0.26, *t*_(3206.2)_ = 258.72, 95% CI [2.38, 2.42], *p* = 2.2*e*-16]. Furthermore, different parameter values of *S* and τ did not affect accuracy, irrespective of which combination was used during the characterization runs. This is also reflected by independent-sample Welch t-tests where the pooled *S* = 1 with the *S* = 5 [*t*_(3, 598)_ = 0.21, 95% CI [−0.07, 0.09], *p* = 0.83], and *S* = 9 [*t*_(3, 597)_ = 0.05, 95% CI [−0.08, 0.08], *p* = 0.96] across all α and τ values were compared and did not show a difference in accuracy. Similarly, when the same analysis was repeated to investigate the effect of τ on accuracy, the results showed that the pooled τ = 0.1 was comparable to τ = 0.5 [*t*_(3597.9)_ = 0.22, 95% CI [−0.07, 0.09], *p* = 0.83], and to τ = 0.9 [*t*_(3597.9)_ = 0.11, 95% CI [−0.07, 0.08], *p* = 0.91] across all α and *S* values. In sum, in trajectories with 50 waypoints, only the α parameter had an impact on accuracy.

### 3.2. Characterization Runs on Trajectories With 100 Waypoints

The same set of analyzes was applied to trajectories with lengths of 100 waypoints. In contrast to the results from Figures 2A,B reveals that α = 0.9 (*M* = 19.31±0.99) led to highest accuracy. To statistically evaluate this, Wilcoxon rank sum tests were used and the difference between different levels of the α parameter was assessed. Again, both *S* and τ were pooled to obtain means corresponding to *M* = 23.69±1.23, and *M* = 21.35±2.26, respectively. A comparison between α = 0.9 and α = 0.5 (*W* = 673730, 95% CI [2.93, 2.96], *p* = 2.2*e*-16), and α = 0.1 (*W* = 92510, 95% CI [1.45, 1.47], *p* = 2.2*e*-16) showed that the difference observed in Figure 2B was statistically significant.

### 3.3. Characterization Runs on Trajectories With 200 Waypoints

In the last set of characterization runs, the highest accuracy was obtained when α = 0.1. Thus, when the strongest corrective feedback was given by the TV-LQR controller (i.e., α = 0.1), the smallest discrepancy between the final system pose and the target grasp pose was observed. As in the case of the previous trajectory lengths, when means were pooled across *S* and τ, it was observed that *M* = 28.71±2.55, *M* = 31.81±2.41, and *M* = 34.59±1.94, for α = 0.1, α = 0.5 (*W* = 466, 280, 95% CI [3.15, 3.16], *p* = 2.2*e*-16), and α = 0.9 (*W* = 75, 702, 95% CI [5.315.33], *p* = 2.2*e*-16), respectively. That is, both statistical tests showed that α = 0.1 had the best accuracy. Notably, Figure 2C also revealed comparable performance in the case where τ = 0.5 and τ = 0.1 (*W* = 1, 589, 900, 95% CI [−0.02, 0.01], *p* = 0.33), but improved performance when compared to τ = 0.9 (*W* = 1, 307, 800, 95% CI [0.07, 0.16], *p* = 2.2*e*-16).

### 3.4. Shared Control Improves Accuracy Across All Conditions

**Figure 3:**
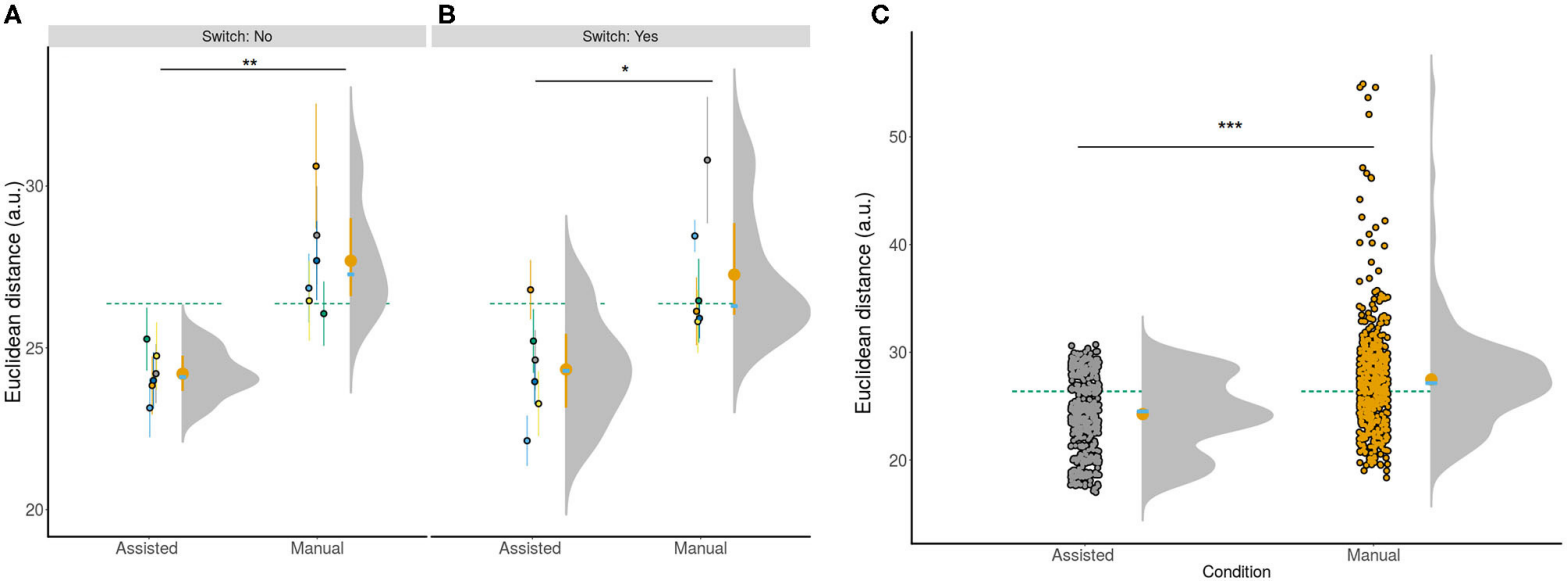
Accuracy of individual conditions for all subjects. **(A,B)** show subject color-coded summarized information (*N* = 6). The accompanying lines denote the 95% CI for the non-switch **(A)** and switch **(B)** condition. **(C)** Shows the same information collapsed across both **(A,B)** in addition to showing raw trial information across all subjects to show the full distribution across all trials. The green dotted line in **(A–C)** is the grand mean collapsed across all conditions. The yellow error bar corresponds to the condition mean and 95% CI. The superimposed blue bar shows the condition median. ***p < 0.001, **p < 0.01, *p < 0.05. All three panels show that the assisted condition resulted in higher accuracy compared to manual control.

With the obtained optimal parameter set, the controller was tested on (*N* = 6) subjects. The main hypothesis was that shared control would improve accuracy compared to manual control. Subjects were more accurate in the shared compared to manual condition (Figures 3A–C) for both the switch [shared - *M* = 24.33±3.51; manual - *M* = 27.26±4.56, *t*_(5)_ = 2.57, 95% CI [0.01, 5.86], *p* <0.05] and non switch [shared - *M* = 24.20±3.41, manual: *M* = 27.69±5.11, *t*_(11)_ = 4.69, 95% CI [1.70, 4.72], *p* <0.001] conditions. Subjects were also more accurate in the shared condition when we collapsed across conditions by computing accuracy estimates per subject [*t*_(5)_ = 4.07, 95% CI [1.29, 5.69], *p* <0.005]. Furthermore, shared control improved accuracy when we investigated the raw data to account for potential outliers in individual trials shared: *M* = 24.26±3.46, manual: *M* = 27.48±4.85) (*W* = 106, 430, 95% CI [2.31, 3.18], *p* = 2.2*e*-16; Figure 3C). These results confirmed that the employed TV-LQR controller outperformed manual control.

**Figure 4:**
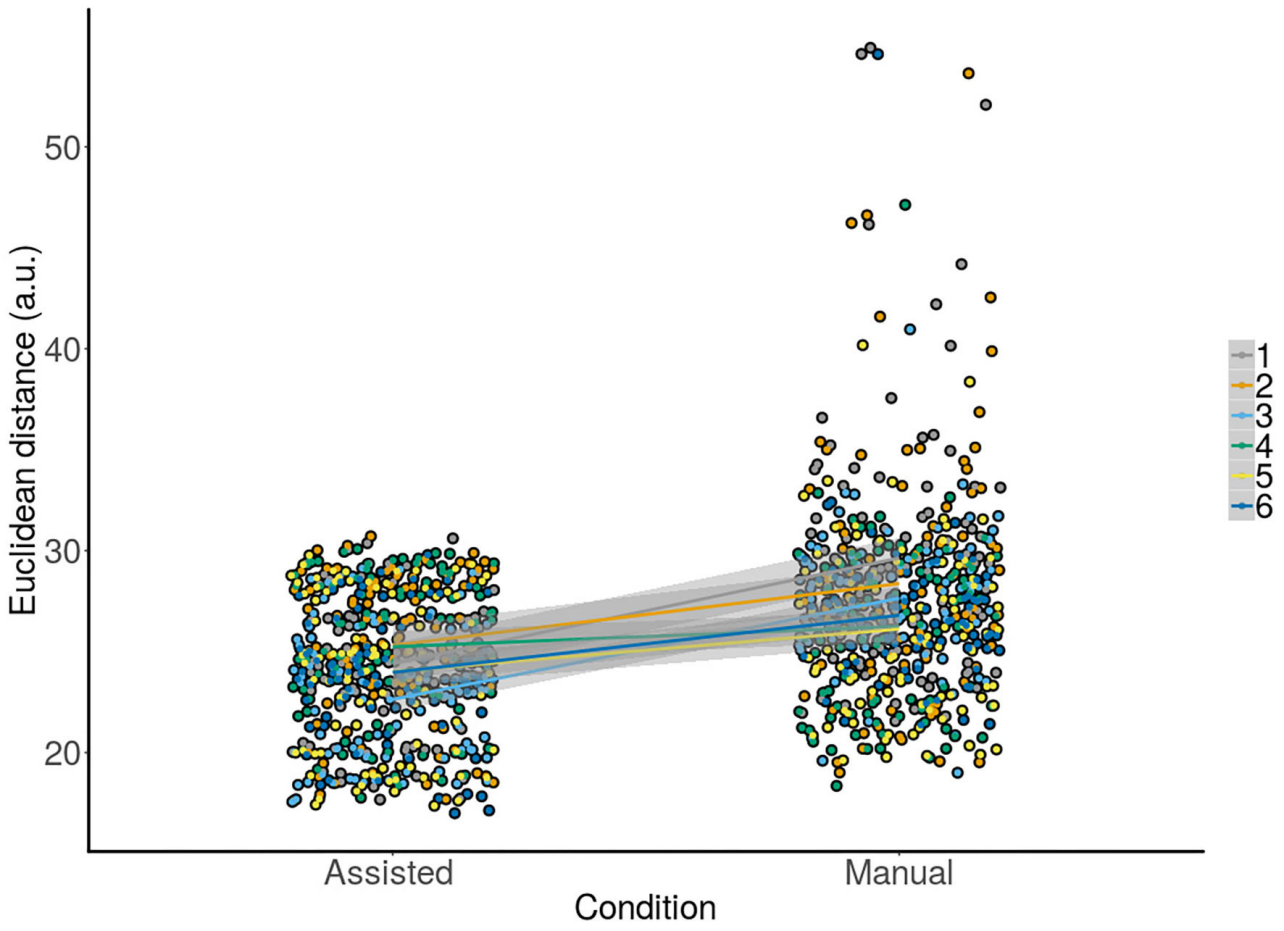
Raw data with corresponding overlaid regression lines and shaded SEs for the assisted vs. manual condition across all subjects. The y-axis shows accuracy for all trials for each subject individually. A positive slope from the assisted to the manual condition means that our controller improved accuracy across trials. This can be observed for all subjects.

Crucially, we hypothesized that the improved accuracy should be observed robustly across subjects as a critical test of the usefulness of our proposed approach. To test, this we modeled the responses of the subject using LMM (Figure 4). We aimed to predict the observed Euclidean distance with the condition (assisted, manual) and information about switching (Yes, No) as fixed factors together with subjects as random intercepts with varying slopes for both condition and switching information, and trials as random intercepts. A Type III Analysis of Variance with Satterthwaite's method showed that only condition [*F*_(5.00, 1)_ = 22.91, *p* <0.005] was a significant predictor for the final observed accuracy or Euclidean distance. A change from shared to manual control resulted in reduced accuracy [β = 3.49 ±0.71, *t*_(6.28)_ = 4.92, *p* <0.005]. In other words, this result implies accuracy deteriorated in the manual compared to the shared condition when accounting for variance in the responses of the subject, in line with our previous results.

### 3.5. Assisted Control Improves Accuracy in All Subjects

**Figure 5:**
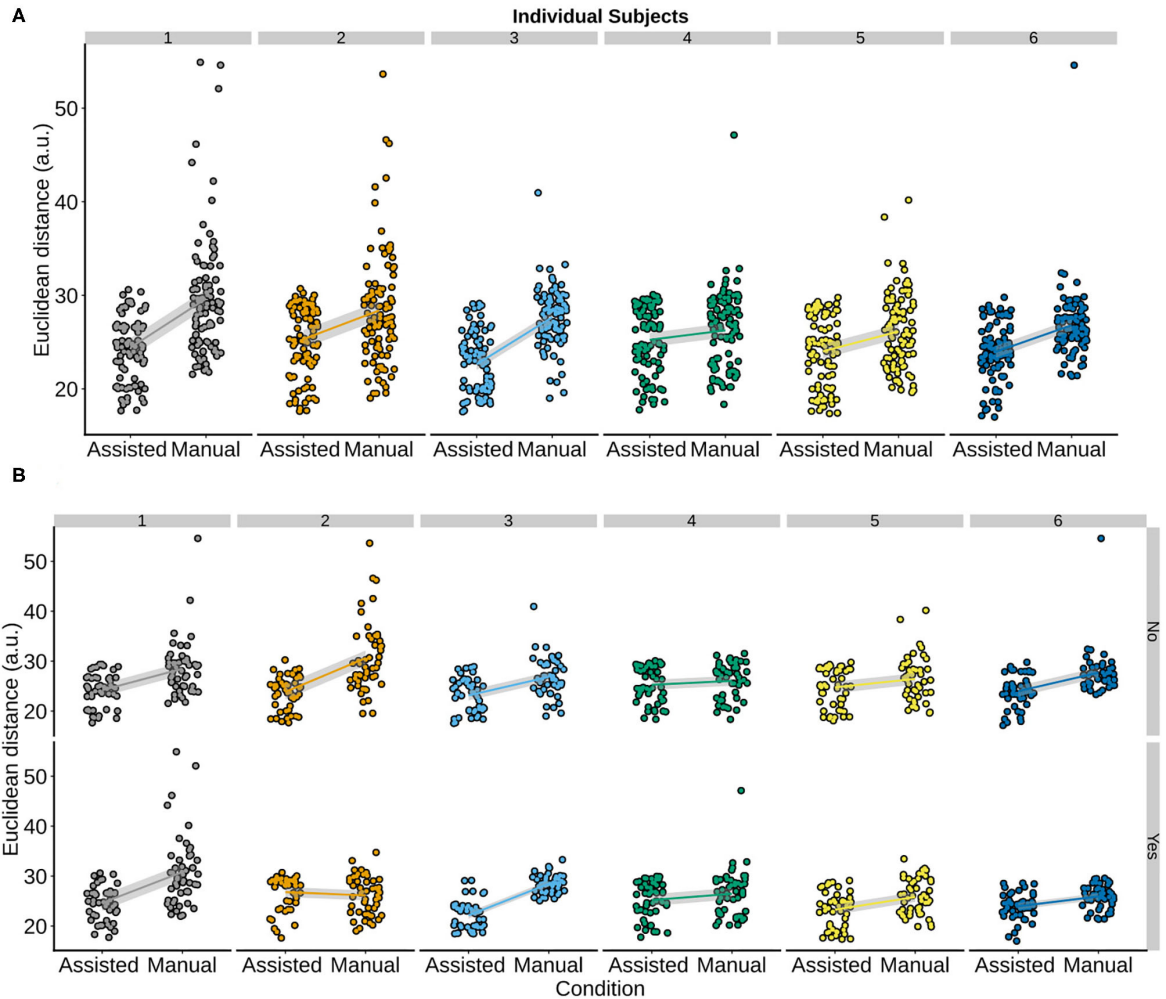
Raw data separated by subject with overlaid regression lines and shaded SEs for the assisted vs. manual condition. The y-axis shows accuracy for all trials for each subject individually. A positive slope from the assisted to manual condition means that the controller improved the accuracy of the subjects. A positive slope can be observed for all subjects in panel **(A)** data were collapsed across the switch and the no switch condition. Similarly, when the data was split according to the switch and no switch condition, a positive slope can be observed for all subjects and conditions in **(B)**, except for the bottom row of the second panel.

As the most stringent criterion for determining whether the controller was better compared to manual control, the conditions were compared for each subject separately. Namely, the focus of this study was to see whether for each subject individually, enabling shared control on a task would yield an improvement in their accuracy compared to manual control where they received no assistance. This is because a significant result in previous tests could also be obtained if shared control improved accuracy for most subjects. To test this, we assessed whether the regression lines for each subject showed a positive slope (β) from the assisted to the manual condition. A positive slope would in this case mean that there was a deterioration in performance when going from assisted to the manual conditions and thereby a general improvement under the TV-LQR assisted condition. We indeed observed (Figure 5A) the βs for individual subjects (*M* = 3.21, *Min* = 1.02, *Max* = 5.23) were positive, showing the assisted condition improved accuracy in all subjects. Similarly, when subjects were further split according to the switch and non-switch condition (Figure 5B), we again observed positive slopes indicating improved accuracy during shared control in all cases except one subject in the switch condition.

In sum, all the described tests that assessed the difference between shared/assisted and manual control provided congruent evidence for our hypothesis of shared control, where the TV-LQR provided assistance, improving the accuracy of the subjects on our task compared to manual control.

**Figure 6:**
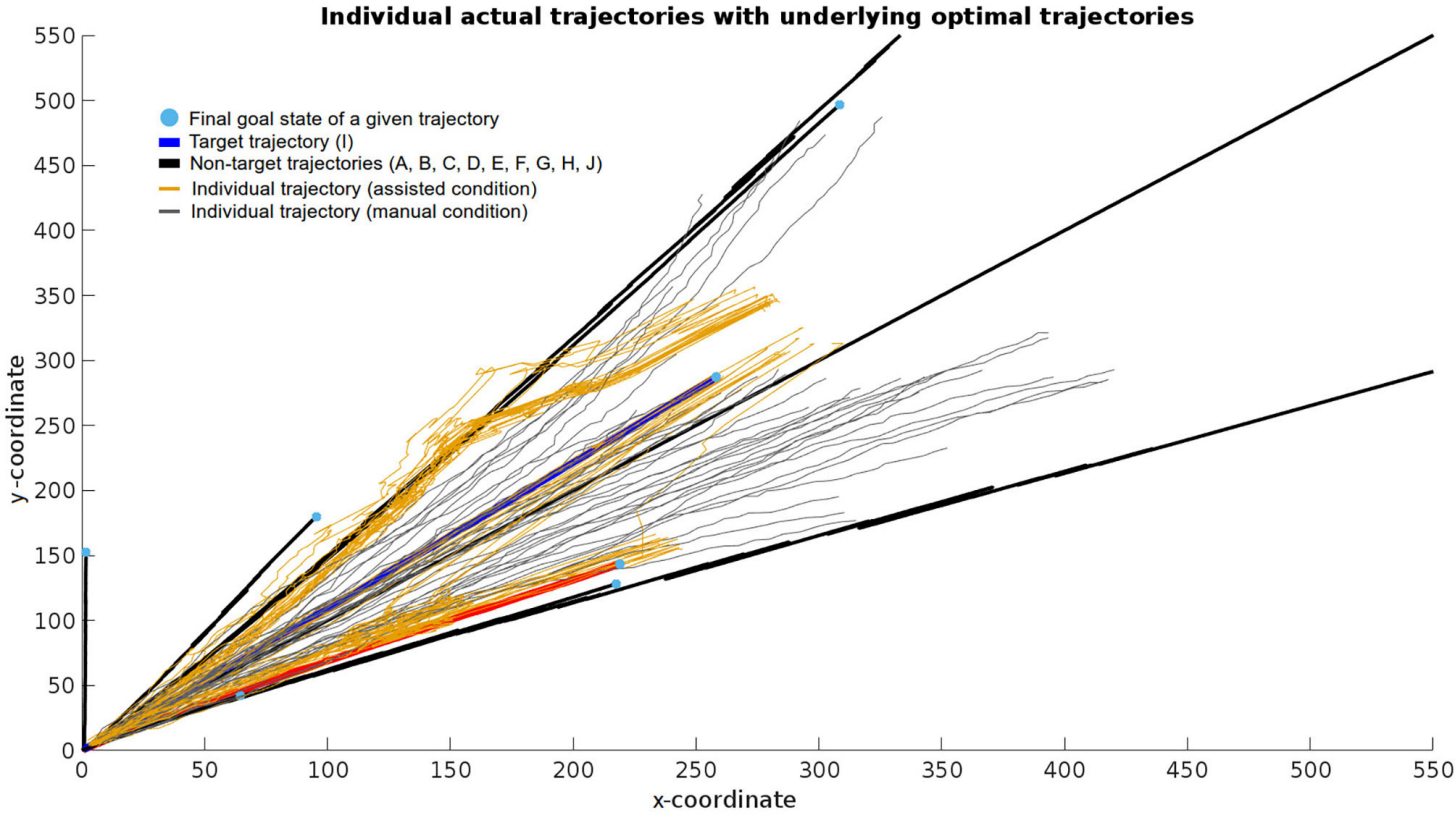
Example data from a series of executions of the manual (gray lines) and assisted (orange lines) condition. Black, red, and blue lines correspond to the optimal trajectories leading to their respective target grasp poses. More specifically, the blue line corresponds to the target trajectory (I), the red line to a neighboring trajectory (F) to which the assisted condition incorrectly converged in some cases, and the black lines to remaining neighboring trajectories. This data displays the starting and ending positions together with the error and dynamics of how the controller handled user input from the beginning until the end of a given trial.

All trials for one example subject were plotted in two dimensions for clarity purposes (Figure 6). For a bundle of runs in the assisted condition, the algorithm first filtered an incorrect trajectory until approximately 3/4 of the trial, after which it started converging on the correct target trajectory. A similar pattern is observable on a few trials where filtering first favored the incorrect trajectory D but then shifted toward the correct target trajectory I toward the end. Furthermore, this figure showcases the inaccuracy of the manual compared to the assisted condition given the goal in both conditions was identical.

### 3.6. Target Trajectories Are More Often Achieved in the Switch Condition Under Assisted Control

**Figure 7:**
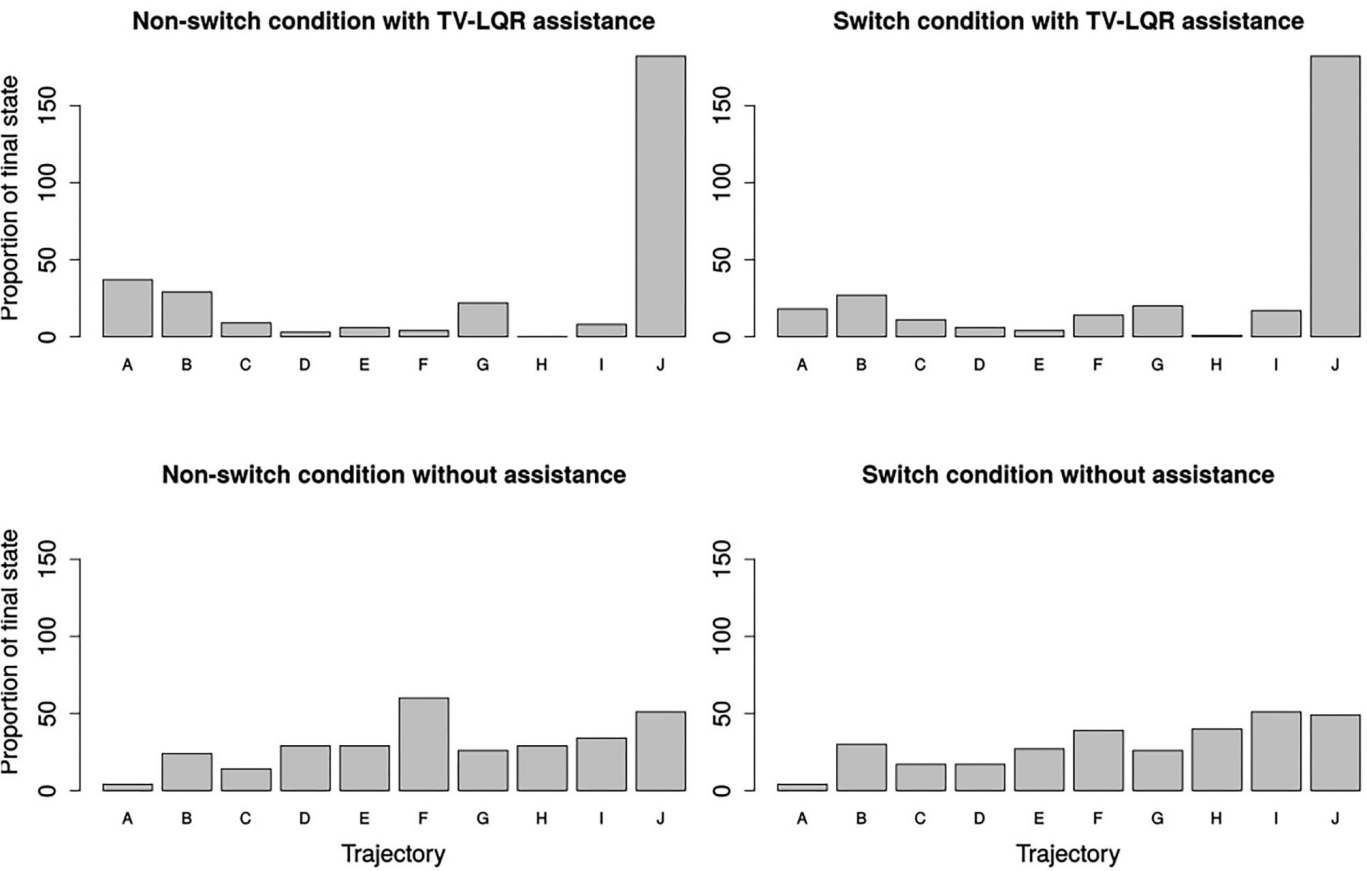
Histogram with the frequency of the final system pose being closest to the target grasp pose. In the case of a random visitation frequency expected by chance, each target grasp, denoted by its trajectory name, would be frequented between five and six times (50 trials per condition).

The last part of the results was related to the prediction of intention. Figure 7 showed a higher convergence toward one of the target trajectories in the case of TV-LQR assistance (upper row, accuracy for non-switch condition: 27.33%, switch condition: 32.33%) as opposed to manual control (bottom row, accuracy for non-switch: 30.33%, switch condition: 30.00%), regardless of whether switching was required or not. Furthermore, in both the manual non-switch (51.67%) and switch (53.67%) conditions, the proportion of non-target final trajectory states (all trajectories except F, I, J) was higher when compared to both the non-switch (35.33%) and switch (29.00%) case of the shared control condition with TV-LQR assistance, indicative of a higher convergence on the final target goal states and better estimation of the proximity of the desired target grasp poses, as picked by the subjects. This shows that, in addition to differences in accuracy between the two conditions, the shared control condition with TV-LQR assistance provided feedback for irrelevant goal states substantially less often in both the non-switch (16.33%) and switch (24.67%) condition.

## 4. Discussion

The presented approach shows how the accuracy and, thereby, the usability of semi-autonomous robots for reach-to-grasp tasks, such as human-operated manipulators for nuclear waste disposal, can be improved. The key idea of the proposed study is to develop systems that are simultaneously context- and user-aware. Context-awareness was implemented as a black box by manually generating feasible grasp candidates and noisy trajectories which served as input to a TV-LQR controller. User-awareness was implemented by filtering of the TV-LQR controller when the user supplied motion commands. We showed the usefulness of the TV-LQR by performing several tests where the grasping accuracy of the subjects was improved with the help of our approach both when they had to or when they were not required to switch between their target grasps.

Our proposed approach employs a predict-then-blend approach, in which the most likely intention of the user is estimated first and then assistance is provided in the tested task. Our semi-autonomous controller takes as input the command of the user at each time step and filters them according to its most likely interpretation of the intention of the user. No switching thresholds need to be defined nor do complex user- or task -dependent functions need to be learned. Our system only requires as input a list of the targets of the user, i.e., feasible grasps and associated trajectories. For such a procedure, we build upon our latest developments on autonomous robot grasping (Kopicki et al., 2014; Zito et al., 2019), which can provide a representation for clutter scenes, generation of feasible grasps, and obstacle-free motion planning for reaching such grasps. However, the work proposed in this study is independent of the way the input is generated, and improving the perception and planning abilities of the autonomous system is out of its scope. In contrast, we present a set of results for establishing the validity of our method in terms of better accuracy in reaching tasks and predicting the user intention. We argue that our TV-LQR controller empowers the user by achieving superhuman performance in terms of accuracy when driving the system to its chosen target. One observation we did not predict was that total accuracy across both the switch and non-switch conditions was similar. This may have occurred due to the employed testing scenario and may be explored in our future study.

In addition to improving accuracy, the system was capable of recovering from incorrect predictions, i.e., when the selected target grasp was not the same as the one chosen by the user. This is best observed in Figure 6, where a bundle of trajectories, which first followed an incorrect optimal reach-to-grasp trajectory, started shifting toward the one the user picked (blue line), and finally converged on that one.

Crucially, because the aim was to build a robust controller system that would mimic real-world use cases of remote tele operations, all the trajectories that were generated had added noise. Similarly, all the tests reported in the results were also performed with non-additive, noisy updating of the system at each waypoint of each trajectory. The main reason for these design decisions was to ensure that the controller would generalize to real-world noisy scenarios well, as both would be predicted when observations are made from incomplete information, e.g., in nuclear waste disposal scenarios and subsequent faulty odometry reading that would render such a system less useful.

Our simulated testing scenario has several advantages for the tests reported in this study. Their simplicity allowed for full control of the environment and a safe test of characterization runs. Moreover, due to this, it was possible to assess the benefits of adding context- and user-awareness with respect to manual control by mimicking a real state-of-the-art setup without potential confounds that could have affected the proposed comparison. Namely, setups in real-world settings require months of training for a human operator to become proficient in a high DOF setting on one or several 2D displays. This is due to the rudimentary and contra-intuitive interfaces employed, i.e., manual control in joint or Cartesian space, and the lack of depth perception provided by a limited number of fixed cameras on the robot site. In contrast, the employed experimental setup circumvented this potential problem to focus and test the proposed framework on naive users after a few training trials. This was due to the fact that the employed experimental setup did not require the users to learn a complex 2D-to-3D mapping between the 2D feedback of a computer display and the 3D environment while providing a full visual description of the scene.

Overall, the results show that using a TV-LQR with a predictive formulation can be used to improve performance on reaching tasks in terms of better accuracy due to robust prediction of movement intention and in terms of being able to recover from incorrect predictions in an online fashion. This was achieved in the simulation where both the controller and the environment separately included noisy components as a proof of principle test for the proposed controller. Furthermore, the importance of parameter tuning was demonstrated in the first part of the results as an auxiliary component when it comes to optimizing such a system. Namely, the employed parameter combination will potentially impact the total accuracy of such controllers. This aspect will become increasingly important as personalized controller systems will need to account for interindividual variability of human users in terms of their motor capacity and control characteristics (i.e., the same default parameter combination might not be optimal for every user), a notion that has been long-acknowledged in other fields (e.g., personalized medicine Schleidgen et al., 2013). In sum, we have shown that the TV-LQR with a predictive formulation is a promising approach that can be used in grasping scenarios with several possible grasp targets. This study paves the way to future implementations where shared control can be assessed in real-world pHRI settings.

## Data Availability Statement

The original contributions presented in the study are included in the article/Supplementary Material, further inquiries can be directed to the corresponding author/s.

## Ethics Statement

Ethical review and approval was provided by the Ethics Committee of Imperial College (ethics approval number 18IC4685). Written informed consent was obtained from all participants prior to them taking part in the experiment.

## Author Contributions

CZ conceived of the presented idea. CZ and SV developed the theory. SV and DF developed the framework, performed and designed the data collection paradigm, the data analyses, and drafted the manuscript. CZ and DF verified the analytical methods. All authors discussed the results and contributed to the final manuscript.

## Funding

This study was supported by the European Research Council Synergy Project Natural BionicS (contract # 810346 to DF). All data needed to evaluate the conclusions in the study are present in the study and/or the Supplementary Materials. Additional data related to this study may be requested from the authors.

## Conflict of Interest

The authors declare that the research was conducted in the absence of any commercial or financial relationships that could be construed as a potential conflict of interest.

## Publisher's Note

All claims expressed in this article are solely those of the authors and do not necessarily represent those of their affiliated organizations, or those of the publisher, the editors and the reviewers. Any product that may be evaluated in this article, or claim that may be made by its manufacturer, is not guaranteed or endorsed by the publisher.
